# Different Conformations of Phosphatase and Tensin Homolog, Deleted on Chromosome 10 (PTEN) Protein within the Nucleus and Cytoplasm of Neurons

**DOI:** 10.1371/journal.pone.0018857

**Published:** 2011-04-29

**Authors:** Vera L. Moncalero, Roxana V. Costanzo, Claudia Perandones, Martin Radrizzani

**Affiliations:** 1 Laboratorio de Neuro y Citogenética Molecular, Centro de Estudios de Salud y Medio Ambiente, Universidad Nacional de San Martín – CONICET, Buenos Aires, Argentina; 2 Fundación Instituto Leloir, Buenos Aires, Argentina; 3 ANLIS (National Agency of Laboratories and Health Institutes of Argentina), Dr. Carlos G. Malbrán, Buenos Aires, Argentina; 4 Parkinson's Disease and Movement Disorders Program, Hospital de Clínicas José de San Martín, University of Buenos Aires, Buenos Aires, Argentina; St. Georges University of London, United Kingdom

## Abstract

*PTEN* is a critical gene involved in the regulation of many cellular processes. The product of this gene has dual phosphatase activity and is able to dephosphorylate the 5′ end of the phosphatidylinositol (3,4,5)-trisphosphate. Within the cellular nucleus, this protein has been associated with regulation of the expression of many genes, although the mechanism of this regulation remains unclear. In this paper, two specific oligonucleotide aptamers were developed and selected, using the SELEX procedure, according to their ability to detect the PTEN protein in different subcellular compartments of neurons. While one aptamer was able to detect PTEN in the nucleus, the other recognized PTEN in the cytoplasm. The recognition pattern of PTEN by both aptamers was confirmed using antibodies in western blots of the proteins purified from mouse cerebellar homogenates and subcellular fractions. Additionally, we demonstrated that the two aptamers recognized different epitopes of the target peptide. The results presented here could not be fully explained by the canonical phosphatase structure of PTEN, suggesting the existence of different conformations of phosphatase in the nucleus and the cytoplasm.

## Introduction

Phosphatase and Tensin homologue, deleted on chromosome 10 (*PTEN*) is a tumor suppressor gene that is deleted in many human tumors and several brain disorders. By deletion of *PTEN* in the mouse central nervous system (CNS), researchers have demonstrated its role in controlling cell migration, size and number. PTEN is also implicated in Lhermitte-Duclos disease, macrocephaly and autism disorders in humans [1].

PTEN contains a sequence motif that is highly conserved among the members of the protein tyrosine phosphatase family and this enzymatic property was suspected to be responsible for tumor suppressor activity, due to the fact that many oncogenes induce tumoral activity by dysregulation of the tyrosine kinase enzyme. PTEN also has phosphatase activity on phosphoserine and phosphothreonine-containing substrates [2], [3] *in vitro* and *in vivo* [4], [5]. Weak dephosphorylation of protein substrates by its tyrosine- phosphatase activity has been observed, and few targets have been identified. In 1998, Jack Dixon et al. demonstrated that PTEN could dephosphorylate the acidic phospholipid, phosphatidylinositol-(3,4,5)-trisphosphate (PIP3) [6] . PIP3 is produced by phosphoinositide 3-kinase (PI3K), which is important for insulin receptor signaling pathways. These signaling pathways produce cytoskeleton rearrangements in the inner cell membrane, driving cell proliferation, migration, differentiation and survival [1]. Spontaneous mutations in the *PTEN* phosphatase domain are oncogenic. PTEN also has a phosphatase-independent activity on tumorigenesis, which may be more tissue-specific or associated with a more aggressive phenotype upon loss of *PTEN* function [7]. Also, phenotypes caused by *PTEN* mutations are not completely recovered by mutations of Dp110 or insulin signaling in *Drosophila* [5], or in mouse models of mammary tumorigenesis [8], suggesting that PTEN may have a broader role than simple antagonism of PIP3.

In addition to its known function in the inner cell membrane, PTEN is also found in the neuronal nucleus [9]. Cytoplasmic PTEN has a well known role as a negative regulator of the PI3K/AKT pathway. It is becoming clear, however, that the role of cytoplasmic PTEN is not the same as that of nuclear PTEN. Nuclear localization of PTEN contributes to its tumor-suppressor activity in several ways. Nuclear PTEN has a role in chromosome and cellular stability, DNA repair, and cell cycle arrest. There is abundant evidence implicating the nuclear protein phosphatase of PTEN in downregulation of the mitogen-activated protein kinase pathway, as well as cyclin D1, and in the induction of G_1_ cell cycle arrest. It has also been observed at the molecular level that the regulation of p53 activity and stability by PTEN is performed via direct protein–protein interactions, independently of its phosphatase activity [10], [11]. Given the fact that many components of the PI3K pathway are also found in the nucleus [12], it is possible that PTEN may be a nuclear PIP3 phosphatase. However, a recent study suggests that nuclear PTEN does not dephosphorylate the nuclear pool of PIP3 [13]. The mechanisms of action of PTEN in the nucleus have remained unclear until now [9].

PTEN phosphatase activity is located at the amino terminal domain of the protein, showing a highly conserved structure and low antigenicity [14]. As a consequence, early observations of nuclear PTEN were believed to be artifacts of poor quality antibodies used in immunohistochemical staining [10]. However, the latest immunocytological and immunohistochemical results with newly developed PTEN antibodies have confirmed the presence of nuclear PTEN in neurons [15] and in a variety of normal [16], [17], [18], [19] and tumor cells [20], [21]. Despite the lack of classical nuclear localization signal (NLS) motifs on PTEN [19], [22], [23], recent studies have shown that several mechanisms could modulate its subcellular localization. These molecular mechanisms include a sequence for cytoplasmic localization signaling [24], PTEN phosphorylation and involvement of Ras-related nuclear protein (Ran)-GTPase activity [25], interaction with major vault protein (MVP) via a bipartite NLS [9], [22], mono-ubiquitination [19], S6K-mediated export [26], and passive diffusion [25]. Recent studies performed by Charis Eng et al. have demonstrated that depletion of ATP results in an increase in nuclear PTEN in various cell lines, suggesting that PTEN subcellular localization is modulated by ATP levels [27]. Specifically, PTEN is predominantly localized in the nucleus of primary, differentiated, and resting cells, as opposed to rapidly cycling cancer cells, where in many cases there is a marked reduction in nuclear PTEN [15], [16], [28]. Therefore, it is surmised that PTEN protein localization may be dependent on both the cell cycle stage and the differentiation stage. This new knowledge begs the question: which function can be attributed to the nuclear PTEN pool?

Furthermore, molecular discrimination between the nuclear and cytoplasmic PTEN proteins may provide an important tool to discern the different functions attributed to the *PTEN* gene. An alternative method for protein recognition can be found in a class of ligand molecules called aptamers, from Latin “aptus” meaning “fit.” Aptamers are high-affinity ligands for a specific target, which are selected using *in vitro* procedures from a combinatorial library. Such aptamers are usually composed of oligonucleotides [29], [30]. They can bind to the active site of a protein or enzyme, thereby blocking its activity. The process of obtaining aptamers is not restricted by antigenicity. Moreover, the nature of their phosphodiester backbones confers high discriminant sensitivity for different protein folding patterns [31]. Taking into account these advantages, we decided to evaluate whether the PTEN protein has different nuclear and cytoplasmic conformations through the use of aptamer technology.

## Results

### Improvements on the combinatorial library of oligonucleotides for SELEX

The most commonly used *in vitro* procedure for obtaining aptamers is systematic evolution of ligands by exponential enrichment (SELEX) [30]. The aim of our research was to obtain aptamers that were able to recognize the native structure of PTEN protein in histochemistry assays, and discriminate different subcellular localizations. For this purpose, some modifications were introduced to the SELEX procedure in order to obtain aptamers with specific binding properties for a large number of possible conformations of PTEN protein.

**Figure 1:**
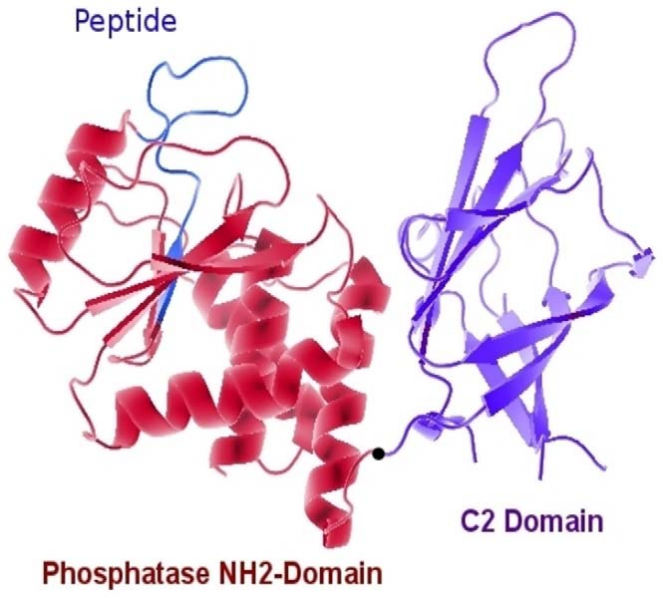
Scheme of PTEN structure. A representation of the PTEN structure (PDB: 1DR5) was obtained by means of the PyMol program. PTEN domains corresponding to the phosphatase domain (PTPs) and the regulatory domain (C2) are displayed in this graphical representation. The peptide selected as target was contained on the PTP domain and was shown in blue color.

The quantity of the oligonucleotide molecules that we used was optimized in order to maximize the amount of aptamers available for binding to the target protein, resulting in a total of 4×10^13^ oligonucleotides. Quadruplicate assays were performed, including modifications of the combinatorial library quantities whilst maintaining the optimum number of total oligonucleotides used for each assay. The oligonucleotide library was modified through amplifications of random aliquots of the whole combinatorial library, and their end effect was measured by assessing the net amount of oligonucleotides bound to target immobilized with nitrocellulose. Maintaining the same concentrations, both the control and treated oligonucleotide libraries were incubated either with the synthetic peptide (**Fig. 1**) or the recombinant protein glutathione-S-transferase/PTEN phosphatase domain (GST-PTEN). The target protein was denatured, placed on a 0.125 cm^2^ surface of nitrocellulose membrane, and incubated using a volume of 300 µl of oligonucleotide library in phosphate-buffered serum-bovine serum albumin (PBS-BSA). Quantities of bound oligonucleotides were measured by means of real-time polymerase chain reaction (PCR), showing an increasing number of bound aptamers after ten or more cycles. Twenty-five cycles of PCR were used as the upper limit, as nonspecific background amplification occurred after the established cycle.

**Figure 2:**
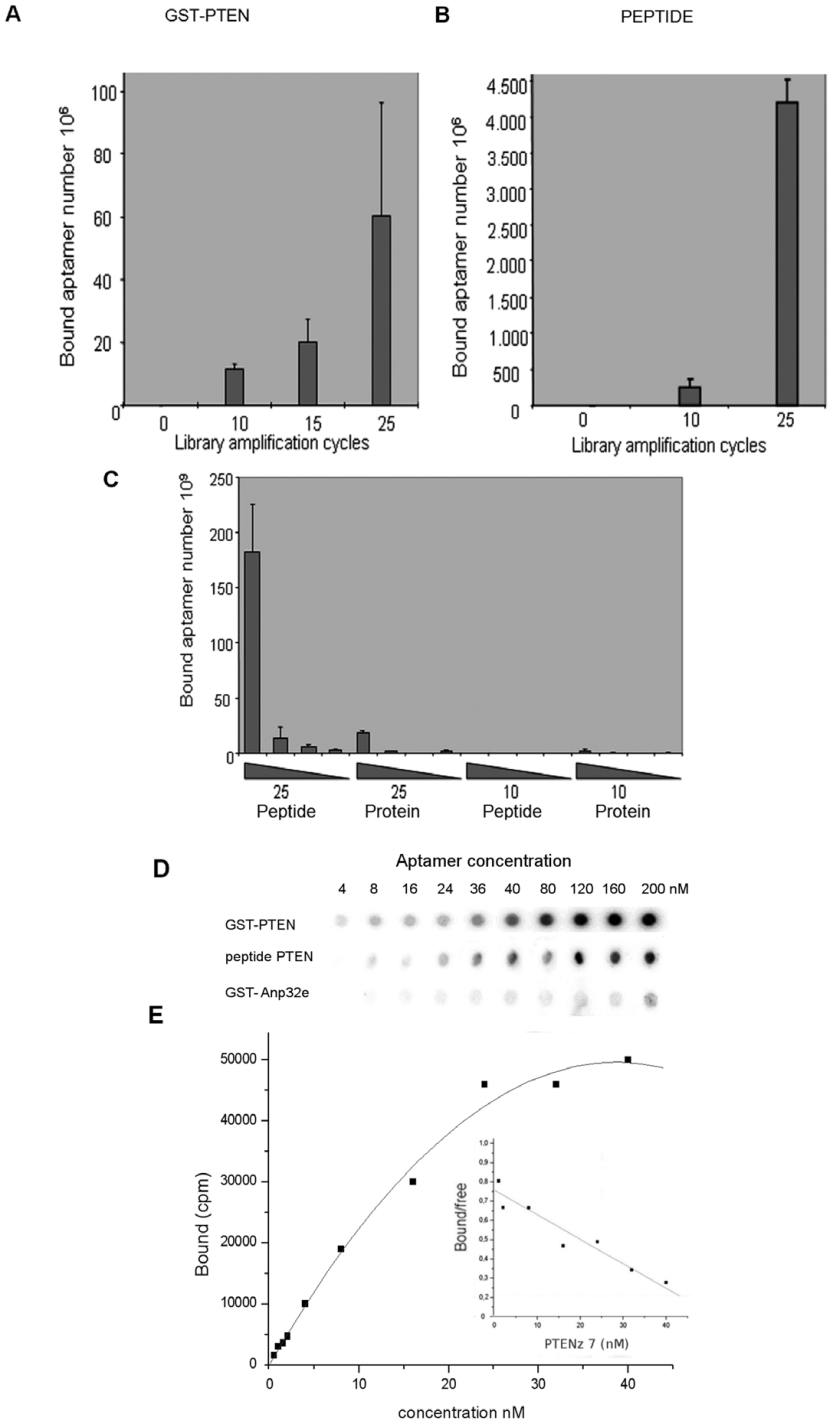
Aptamer's selection using PTEN recombinant protein and PTEN synthetic peptide. **A**. In these assays, selected oligonucleotides were separated from the GST-PTEN target, indicated above the graph, and quantified by means of real-time PCR. The effect of amplification of the library on the quantity of oligonucleotides obtained is illustrated in this graph. The number of molecules, as indicated on the y axis, is obtained through extrapolation of the standard curve, while the number of cycles applied to the library used is indicated on the x axis. The three assays show a significant increase compared with the library without amplification, used as control. Libraries were amplified through PCR separately for each assay. Standard deviation is indicated using bars on each column. **B**. Results for assays as described above, using the synthetic peptide of PTEN as target, instead of GST-PTEN. Libraries were separated in aliquots from a larger pool that was amplified through PCR. **C**. Specificity of the oligonucleotides obtained. Aliquots of the selected oligonucleotides were amplified again through PCR, separated, and split into four dilutions. The filter binding assays were performed with different concentrations for four separate selections of aptamers, and each selection is indicated at the bottom of the graph. The gradient of concentration follows the slope of the triangle and the standard deviation is indicated using bars on each column. **D**. Affinity of the aptamers obtained. Autoradiography of radioactive aptamers bound to specific targets. The aptamer PTENz7 recognized the recombinant PTEN protein and the peptide without binding to the GST-Anp32e recombinant protein in filter binding assays. **E**. Selected oligonucleotides developed with 25 cycles of library amplification, using the synthetic peptide of PTEN as target, were cloned into vectors and sequenced. Plasmids from six different clones were labeled by PCR containing ATP-gamma-^35^PO_4_, incubated, and their affinity was measured in Scatchard binding assays. The example shown is the oligonucleotide from clone PTENz7, and the affinity constant measured corresponds to 75 nM, using GST-PTEN protein at a final concentration of 25 µM. At the bottom of the graph the autoradiographic results from the radioactive aptamers retained in the filter binding assays are shown. The concentration of the aptamers used corresponds to the measurements illustrated within the graph. On the x axis, the concentration of PTENz7 in nM is provided. On the y axis, the ratio between bound and free aptamers is shown.

The results, obtained after performing the process in quadruplicates (**Fig. 2A**), indicated that the control library has substantial background noise, showing the need for amplification. The figure also illustrates that the number of bound oligonucleotides obtained in the abovementioned conditions increased significantly with the application of further cycles, although a specific correlation was not established.

As shown in **Fig. 2B**, the same results were obtained using peptide instead of GST-PTEN as a target. It was also observed that, in comparison with GST-PTEN, the amount of bound peptides was several magnitudes larger. The reason for the difference can be explained by the difference in size between the recombinant protein and the peptide. The smaller size of the peptide allowed a larger amount of molecules to stick to the nitrocellulose. When the two molecules were compared, the ratios between size and number of bound aptamers was the same, supporting the explanation provided above. Another interesting observation using the peptides was a notable decrease in their variation, caused by a difference in the methods used in the two series of experiments. For GST-PTEN, four separate libraries were prepared and amplified; in contrast, for the peptide, only one library was prepared and amplified, and then split into four separate aliquots. The latter method achieved a more homogeneous library, resulting in less variation.

The next step was to confirm the recognition of the GST-PTEN by the selected aptamers obtained through targeting of the peptides and recombinant protein. Selections were made with 10 and 25 cycles of PCR (**Fig. 2A** and **Fig. 2B**). Given that each selection was done in quadruplicate, the selected library with the best results was chosen. Through the use of filter binding assays, we confirmed the specificity of the aptamers to the target protein and the affinity of the selection. The test compared four separate groups of aptamer libraries obtained from the previous assays. The chosen groups are as follows: library with 25 PCR cycles using target protein; library with 25 PCR cycles using target peptide; library with 10 PCR cycles using target protein and library with 10 PCR cycles using target peptide. Within each group, four different dilutions of aptamers were used for four filter-binding assays. As shown in **Fig. 2C**, it was clear that the library obtained with 25 cycles of PCR using target peptides also recognized the recombinant protein. That result could be concluded from the filter binding assay with the highest concentration of aptamers available, since there was a large number of bound aptamers. We noted that the number of bound aptamers decreased drastically with the dilution of the aptamer solution, indicating that the affinity of the library was near the range of the working dilution. Because the library was made up of many different aptamers, this observation indicated a standard behavior.

Finally, aptamers isolated from both selections were cloned, sequenced and their affinities were measured by Scatchard analysis. The affinity for the studied aptamer, PTENz7, was found to be 75 nM as shown in **Fig. 2D**, and PTENz14 affinity was 54 nM (data not shown). Other aptamers had affinities ranging from 50 nM to 120 nM (data not shown). Thus, the aptamers obtained through amplified libraries were specific to GST-PTEN and showed the affinity required for our studies.

### Analysis of the oligonucleotide aptamer candidates by western blot assay

**Figure 3:**
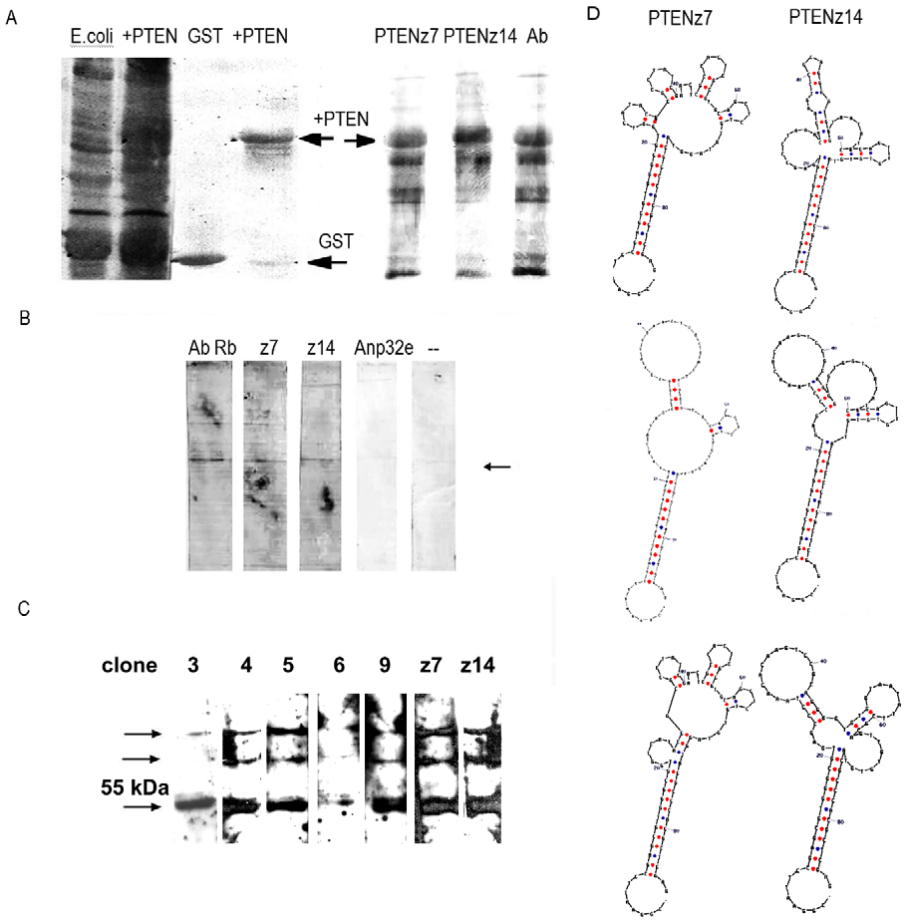
PTEN recognition in Western blot assays and predicted PTEN aptamer conformations. **A**. Protein pattern expression in *E. coli* of the recombinant proteins GST (E. coli) and GST-PTEN (+PTEN) in conjunction with the affinity column-purified proteins (GST and +PTEN). The identity was confirmed by binding of the goat antibody (Ab), resulting in similar patterns to the cloned aptamers derived from peptide (PTENz7 and PTENz14). **B**. Western blot using aptamers PTENz7 and PTENz14 (z7 and z14) and antibodies (Ab Rb) developed with peptide in the cerebellar homogenates. The synthetic aptamer strand of Anp32e was used as a negative control, as well as the omission of aptamers (–). The arrow indicates the most intense band corresponding to the 55-kDa PTEN isoform. **C**. Western blot detection made as in (B) using aptamers and antibodies developed against the GST- PTEN recombinant protein. The arrows indicate the band of 55 kDa and two bands of greater size, possibly due to ubiquitin-modified PTEN. D. PTEN aptamer conformations: Sequences from PTENz7 (left column) or PTENz14 (right column) were analyzed for internal interactions and the best three predicted structures (mFOLD, http://www.idtdna.com/Scitools/Applications/mFold) were presented in this panel. For the more stable conformation of PTENz7, a differential free energy of Gibbs of −19.04 kcal.mole-1 was observed and for PTENz14 this value was −17.3 kcal.mole-1.

Aptamers obtained from peptides or from the recombinant protein were cloned, and twenty of these clones were sequenced. The specificity of each aptamer for recognition of the recombinant protein was confirmed by dot blot assays (not shown). The results show different oligonucleotides in the variable region with no conserved sequence motif. These findings were in concordance with a model containing a large diversity of products obtained from the selection step. This is also in accord with our goal of obtaining a large number of aptamers that can recognize a wide spectrum of conformations, in the hope that at least one of these recognizes the protein synthesized and modified in the cerebellum. We proceeded to confirm that the aptamers obtained from the selections performed with peptide also recognized the GST-PTEN and the PTEN protein from cerebellum homogenate using western blot assays. As shown in **Fig. 3A**, aptamers PTENz7 and PTENz14 were effectively capable of recognizing the recombinant protein GST-PTEN. In **Fig. 3B**, the comparison between western blots using aptamers and western blots using antibodies developed against peptide is shown. The similar staining patterns indicated that both the antibodies and aptamers recognized the protein. Similar results were obtained for **Fig. 3C**, where the target, from which the aptamers and antibodies were derived, was the recombinant protein instead of the peptide. The fact that not all the aptamers recognized the cerebellar PTEN protein in western blot assays was perhaps a consequence of its post-translational modifications. The design importance of the potential structures that the aptamers can take is shown in **Fig. 3D**. All structures form the double helix hairpin in the “constant region of the primers”, which is the region involved in providing molecular stability to the aptamers. The resulting structures of the aptamers (obtained through mFOLD, http://www.idtdna.com/Scitools/Applications/mFold) are in concordance with those shown in **Fig. 3D**.

### Analysis of the aptamer recognition properties of PTEN in histochemistry assays

**Figure 4:**
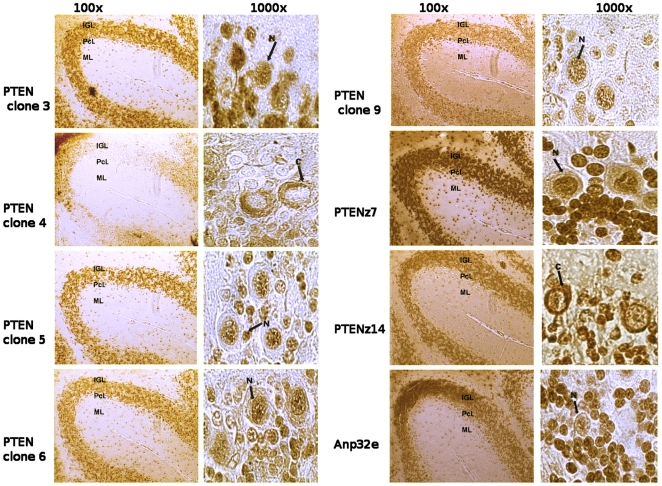
PTEN aptamers and PTEN detection in cerebellar histologies. Staining pattern in histologic samples of mouse cerebellum at two magnifications, 100× and 1000×, as indicated at the top of each column. To the left of each pair of panels the aptamer or antibody utilized for the histological staining assay of the PTEN protein was denoted. The layers of the cerebellum are indicated within each illustration as internal granular layer (IGL), Purkinje cell layer (PcL) and molecular layer (ML). The Purkinje cells are shown at the highest magnification, 1000×, where nuclear (N) or cytoplasmic (C) localization is distinguishable. The known aptamer Anp32e (Anp) was used as a positive control. The patterns belonging to clones 3, 4, 5, 6 and 9 corresponded to the aptamers developed against the recombinant protein GST-PTEN, while the patterns associated with z7 and z14 were the aptamers PTENz7 and PTENz14 developed against the peptides.

Special care was taken with the brain fixation for histochemistry assays, using non-crosslinking fixation to avoid denaturing the protein. An ethanol - acetic acid mixture (95%∶5%) was chosen, because Carnoy's fixative and, more generally, ethanol-based non-crosslinking fixatives have been reported [32] to allow a satisfactory preservation of the antigenicity of proteins and their epitopes in tissues [33], [34]. It may be advantageous to investigate the native distribution of cellular and extracellular components and to compare affinity and specificity of different reagents for a target ligand [35]. Under these conditions, the “naive” PTEN was analyzed in histochemistry assays using the selected aptamers, and subcellular localization was observed in detail (**Fig. 4**).

The two aptamers, designated PTENz7 and PTENz14, were selected according to the following properties: i) The aptamers recognize the same known antigen site of the protein; ii) The aptamers recognize the recombinant and the native PTEN protein whether in a denatured or naive conformation; and iii) The aptamers discriminate nuclear from cytoplasmic PTEN in histologic assays.

The properties analyzed for some aptamers are summarized in **Table 1**.

**Table 1:** Aptamer's properties for PTEN protein detection.

Aptamer	Western blot	Histology	Cellular localization
**Clone 3**	+	++	Nuclear
**Clone 4**	+	+	Cytoplasmic
**Clone 5**	+	++	Nuclear
**Clone 6**	−	+	Nuclear/Cytoplasmic
**Clone 7**	+	−	−
**Clone 8**	+	−	Nuclear
**Clone 9**	+	+	Nuclear
**PTENz7**	+	+++	Nuclear
**PTENz14**	+	+++	Cytoplasmic

Properties measured in the aptamers derived from peptides (PTENz7 and PTENz14) and cloned aptamers derived from the recombinant protein GST-PTEN (Clone 3, Clone 4, Clone 5, Clone 6, Clone 7, Clone 8 and Clone 9). The columns: western blot, histology and cellular localization show the performance of each aptamer for the corresponding assay and their subcellular localization. Aptamers PTENz7 and PTENz14 are recognized as the best choice considering their ability to recognize the native protein PTEN, their well-defined localization and different manifestations in subcellular regions.

### Specificity of the aptamers PTENz7 and PTENz14 evaluated by histological assays

**Figure 5:**
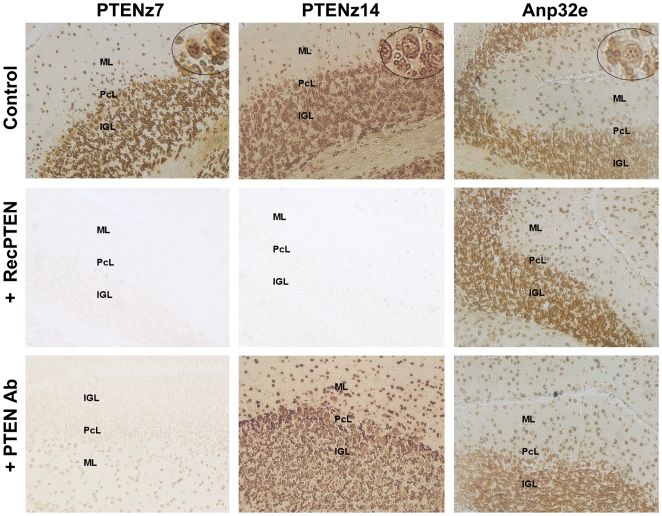
Specificity of PTEN staining in cerebellar histologies. The PTEN staining pattern using aptamers analyzed in cerebellar slices from mice. PTEN was detected with the peroxidase system within the cerebellar cortex of adult mice using the selected aptamers and a positive aptamer control developed from a peptide contained within the Anp32e protein sequence. The aptamer used is indicated in the column heading, while the designated treatment with recombinant proteins or antibodies is mentioned to the left of the rows. The first row shows the histological staining without any competition for the aptamers. The second row indicates aptamers pre-incubated with the recombinant protein before and during the histologic assay. The third row shows histologic samples where aptamers were pre-incubated before and during the histological assays with rabbit antiserum developed against the PTEN peptide. The cerebellar cortex layers are indicated in each micrograph as: Molecular layer (ML); Purkinje cell layer (PCL); Internal granular layer (IGL). Images were obtained at 100× magnification. The subcellular localizations are shown in the upper circle of each aptamer staining in the control row.

The potential artefact of the aptamer staining pattern in the histologic samples was further evaluated through the use of different controls. PTEN recognition was demonstrated by incubation of the oligonucleotide aptamers developed with the synthetic peptide and with the recombinant protein PTEN. The recombinant protein competed with the native PTEN protein for the aptamers, PTENz7 and PTENz14, resulting in a decrease in histologic staining (**Fig. 5**). The presence of the recombinant protein did not affect the staining properties of the aptamer used as control (Anp32a protein). The positive results of this control confirm that the blockage of the aptamers depends on the sequence motif and not on some nonspecific interaction between the DNA and the recombinant protein.

In advance, we had developed a rabbit antiserum against the same synthetic peptide of PTEN [36]. Thus, a control of the aptamers' specific recognition of PTEN within the tissue could be achieved by masking the specific antigen using this antiserum. Histologic samples were pre-incubated with the antiserum and during the incubation with the aptamer. As expected, the staining was poor or absent in the antiserum-treated histologic samples incubated with PTENz7 aptamer, a fact that demonstrates the specificity of the target recognition by the aptamers.

Unexpected results were obtained with aptamer PTENz14, where no alterations of the staining pattern were observed. There are two possible explanations for that finding. The first possibility is that the aptamer recognizes another epitope from within the same antigen of the antiserum, and antibodies are thus unable to block the aptamer–PTEN interaction. The second possibility is that the aptamer recognizes a protein other than PTEN, so the antiserum would produce no detectable changes in the staining pattern.

### PTENz7 and PTENz14 recognize different epitopes from the same antigen peptide

The results presented show that the aptamers PTENz7 and PTENz14 recognize the peptide and the recombinant protein in solution or in the nitrocellulose membrane. This leads us to consider the possibility that the rabbit antiserum is incapable of recognizing the entire peptide used as target. The peptide consists of 14 amino acids, and the adequate length for antigen presentation is about 7–8 mer [37]. Therefore, there are two possible epitopes. It is plausible that, as a consequence of protein conservation during evolution [10], [14], the antiserum recognizes only one portion of the antigen, while the other portion of the peptide is non-antigenic.

**Figure 6:**
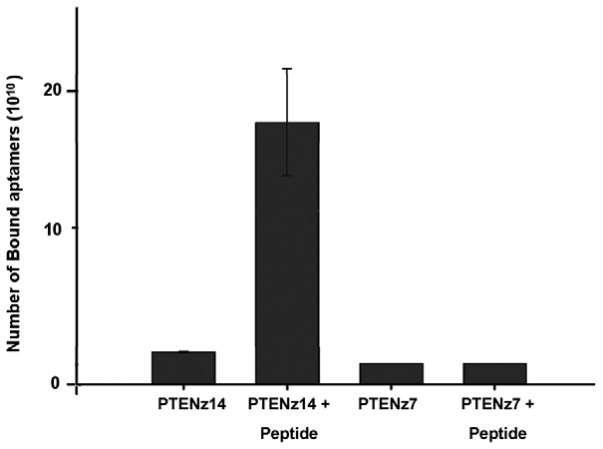
Simultaneous recognition of PTEN peptide by aptamers and antibodies. The results of precipitation are shown in terms of net amounts of molecules acquired through extrapolations from the standard curve of quantification. The backgrounds considered (PTENz7 and PTENz14) were attained through incubation and co-purification of the aptamers and antibodies in the absence of peptides. Quadruplicate samples showed the positive binding of PTENz14 to the peptide (PTENz14+Peptide). Findings also confirmed the blockade of PTENz7 recognition by the antibodies (PTENz7+Peptide). Extrapolations from the standard curve of quantification show 1.8×10^11^ molecules of PTENz14 aptamer were bound to the peptide and co-precipitated with the antibodies attached to Sepharose – nearly a 20-fold increase with respect to the background (P<0.0001).

To test the hypothesis of incomplete recognition of the peptide by antiserum, a specific experiment was performed. If the hypothesis was correct, the antibody would be able to bind to the peptide, hiding the epitope of the aptamer PTENz7, while the aptamer PTENz14 would bind to the other free epitope. Through co-purification of the double-bound peptide, the aptamer PTENz14 and the antibody would precipitate. If the levels of the peptide were increased, the proportion of aptamers measured would increase above the background level. If the recognized epitope was blocked by the antibodies, the aptamers would be unable to bind to the peptide and so would leave the oligonucleotides' background unaltered. The bound aptamer molecules would then be easily detected by means of real-time PCR (**Fig. 6**). The results obtained with the assays of co-purification were consistent with the model of incomplete recognition and explained the lack of interference of the antiserum in tissue staining with aptamer PTENz14.

### Comparison of PTEN detection in histologic samples using aptamers and antibodies

**Figure 7:**
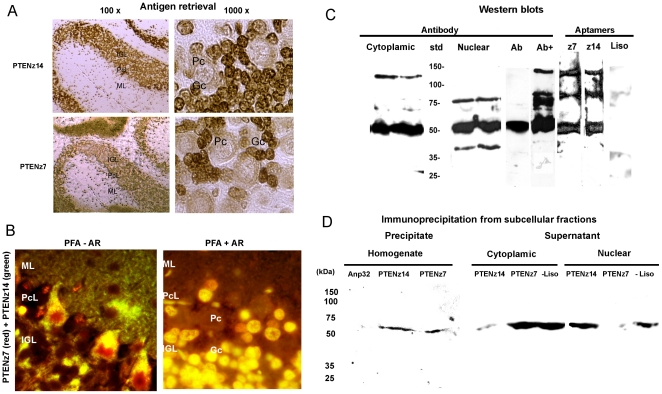
Antigen retrieval and PTEN detection in cerebellar subcellular fractions. **A**. *No crosslinking fixation*. Histologic samples showing the peroxidase staining pattern of denatured proteins. 100× and 1000× magnifications are indicated at the top of each column. The denaturation was achieved through heating of the tissue, a technique commonly known as antigen retrieval (AR). The aptamer utilized for the staining is indicated on the left of each row. The cerebellar cortex layers are indicated in each micrograph as: molecular layer (ML); Purkinje cell layer (PcL); internal granular layer (IGL). The micrograph at 1000× magnification indicates Purkinje cells (Pc) and granule cells (Gc). **B**. *Crosslinking fixation*. Histologic samples fixed with paraformaldehyde (PFA) with and without antigen retrieval (+AR and -AR respectively) were stained simultaneously with PTENz7 (CY3-red) and PTENz14 (FITC-green). Colocalization was observed as yellow labeling at 400× magnification. **C**. *Western blot subcellular fractions and homogenates*. Homogenates and subcellular fractions were developed using a polyclonal antibody against the carboxy terminus of PTEN. Cytoplasmic fraction presented a large amount of PTEN, corresponding to the 55-kDa band. Another band of 120 kDa was also observed in this fraction (duplicates). The nuclear fraction presented a major band of 55 kDa with two minor bands of 80 kDa and 40 kDa. In homogenates, the major band of 55 kDa was observed (Ab), but required overexposure (Ab+) to detect the modified-mobility isoforms. The band of 55 kDa had a lower intensity than the antibody band. Both aptamers PTENz7 (z7) and PTENz14 (z14) detected the 80-kDa and 120-kDa bands. Controls were put in place using the negative chain corresponding to the lysozyme aptamer (Liso). **D**. *Immunoprecipitation assays*. Proteins from cerebellum homogenate were utilized to purify PTEN by means of the aptamers PTENz7 and PTENz14. The aptamer Anp32 was used as a negative control for the purification. The purified proteins were separated in denaturing 10% SDS-PAGE, transferred to a nitrocellulose membrane and detected using goat antibodies against GST-PTEN protein. Corresponding aptamers are indicated above each western blot lane, while the molecular weight is denoted to the left of the image. The PTEN protein was precipitated from the subcellular fractions and the presence of the protein was analyzed in the supernatants. Note that PTENz7 was able to diminish the PTEN staining from the nuclear fraction, whereas PTENz14 affects the cytoplasmic fraction.

If the recognition of PTEN by each aptamer differs due to an alternative conformation of the native protein, then the histological staining pattern with each aptamer – given that the protein is denatured by the heating of the tissue required for antigen retrieval (AR) – is expected to be similar. We observed that, after the denaturation of the protein, histological assays of PTENz7 and PTENz14 effectively resulted in the same staining pattern (**Fig. 7**). Using crosslinking fixation with paraformaldehyde, nuclear staining for both aptamers was absent from the granule cell nuclei and instead colocalized in the mossy fiber synapses. Nuclear staining of the Purkinje cells was restricted to the PTENz7 aptamer, whereas the PTENz14 aptamer was relegated to the cytoplasm, with granular colocalization of PTENz14 and PTENz7 remaining within the Golgi system. Nuclear staining of the granule cells was unmasked when the AR technique was applied to these tissues, and resembled the colocalization results obtained with AR in alcohol/acid fixations. The lack of staining in the Purkinje cells was observed using both kinds of fixations, and could possibly be attributed to the fact that the proteins became soluble and were eluted from the tissues.

The nature of PTEN localization in the nuclei can be attributed to binding by one ubiquitin modification, which also produces a delay in electrophoretic mobility. We therefore analyzed for PTEN isoforms in a gel electrophoresis assay using a polyclonal antibody against the carboxy domain and subcellular fractionations. The results obtained using overexposure of the western blots showed the presence of one ubiquitin modification in the nuclear fraction and two ubiquitin modifications in the cytoplasmic fractions. Comparisons between aptamer and antibody staining in western blot assays are presented in **Fig. 7C**. In this figure, we show that either aptamer was able to recognize PTEN with the ubiquitin modifications, and that this is not the cause of the subcellular discrimination of the aptamers. Note that the intensity of the band corresponding to PTEN without ubiquitin was diminished in both aptamers. It could be possible that neither aptamer recognizes all isoforms of PTEN. As further proof that the selected aptamers were capable of recognizing the native protein, we used “immunoprecipitation” assays, presented in **Fig. 7D**. PTEN was purified from a homogenate protein solution with the selected aptamers. The presence of PTEN was verified through the use of goat antibodies in western blot assays, confirming that each aptamer was able to recognize naïve PTEN in its native conformation. Through an assay with the control aptamer (Anp32) that does not recognize PTEN it can be concluded that the PTEN protein does not display nonspecific interactions with the purification reagents. These results were further supported by interpretation of subcellular fractionation assays and the ability of each aptamer to deplete PTEN specifically from each of the nuclear and cytoplasmic fractions. Taking these findings into account, the aptamers, recognizing different extremes of the peptide sequence, could differentiate protein localized by histological staining or in subcellular fractions. Due to the fact that the peptides were partially included in the phosphatase structure described, these results reinforce the existence of a new conformation of PTEN, different from the known phosphatase conformation that is present in the neuron.

## Discussion

The results presented here showed, for the first time, a different conformation of the amino domain of the PTEN protein in neuron nuclei. Since we did not detect the phosphatase conformation, it is appropriate to surmise that PTEN has a non- phosphatase function in the nucleus.

Our findings are consistent with recent nuclear PTEN studies, which also suggest a non-phosphatase role of this protein, such as regulation of p53 structure and function through direct protein–protein interaction free of phospholipase activity [11], [38]. Furthermore, the latest research demonstrates that PTEN does not influence the dephosphorylation of the PIP3 nuclear pool [13].

Another finding supporting our hypothesis has been described by Goberdhan et al., in which Drosophila phenotypes produced by mutations of *PTEN* are not entirely offset by Dp110 mutations [5].

Also, on a technical note, the results presented here show an improvement on the SELEX method [39]. The oligonucleotides obtained with this procedure have high affinity and specificity for the PTEN protein or a chemically synthesized peptide used as target. In contrast with other improvements on the SELEX method, which use a greater number of selection steps with the native protein [40], [41] or the recombinant protein [42], we used a single-selection step with a preset amplification of the library.

Although it reduces the number of selection steps of the process, our improvement on the SELEX method does require evaluation of a greater number of clones to obtain the aptamer with the desired properties.

There is a large body of evidence suggesting that more aggressive cancers are linked to the absence of nuclear PTEN in patients [16], [17], [21], [43], [44], [45] Thus, nuclear and cytoplasmic detection of PTEN using aptamers and antibodies could be an important indicator for clinical prediction. Our goal was to obtain aptamers that were able to discriminate between nuclear and cytoplasmic localization of PTEN, while giving priority to those directed at a known target sequence.

**Figure 8:**
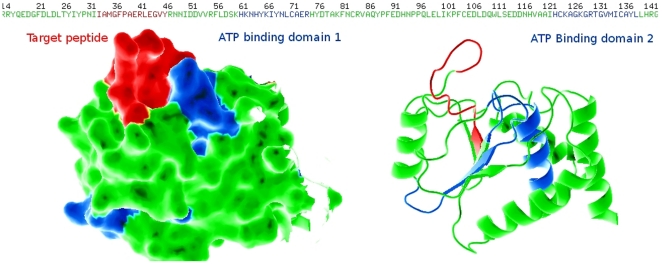
Scheme of PTEN structure showing the selected PTEN peptide and the ATP-binding regions. The figure shows the regions of interest: the amino domain of the PTEN protein surface and the backbone. The peptide surface exposed on the PTP domain is shown in the peptide sequence (detailed in the upper left hand corner). The ATP binding regions are shown in blue, and the target peptide is shown in red as shown in the upper sequence. Note that the ATP binding peptides correspond to the internal portion of the phosphatase structure. The graphic was developed using the PyMol program.

Unexpectedly, the differential recognition of the nuclear and cytoplasmic isoforms of PTEN was due to the recognition, by the aptamers, of different regions of the peptide. The manner in which different epitopes of the peptide were displayed was determined by different conformations of the amino domain of the PTEN protein (**Fig. 8**).

As an additional post-translational modification that occurs in neuron cell cultures, ubiquitin was found in the PTEN protein of the nucleus and two *ubiquitins* were found in the 13 and 124 amino acids of PTEN [46]. Our results describe for the first time the existence of both isoforms of *ubiquitin*-modified PTEN as stable proteins in the nucleus and cytoplasm of cerebellar neurons. Our aptamers showed greater preference for the ubiquitin modified PTEN than the non-*ubiquitin* modified PTEN in western blot assays. Precipitation assays showed the ability to precipitate the majority band, but not all of the protein present was depleted.

Recent studies demonstrate the effect of ATP on the localization of nuclear PTEN. The authors found two sites of ATP binding that correspond to the 60–73 and 122–136 amino acids of the phosphatase domain [47]. Our target region, corresponding to the 31–43 amino acids of the second beta-sheet of the same domain and, as in ATP domains, 60–73 and 122–136, presented only a small region exposed to the surface with a large part in the inner structure of the phosphatase. A change of conformation of this structure was expected for a switch of the PTEN localization and function into the nuclear environment (**Fig. 8**).

One part of the utilized peptide is recognized by rabbit antibodies and the other part of the peptide has the capability of being recognized only by one of the aptamers. According to these results and assuming that the aptamers recognize both extremes of the peptide, one of them must be located internally in the protein structure. For this internal extreme to be exposed and detectable by the aptamer, the protein would have to be open or with a fold different to the description for the structure of the phosphatase. Our hypothesis that there exists a relevant structural change which establishes the exposure of different regions of the PTEN protein is also supported by the discoveries of the group of Dr Eng [27], [47]. Such results, obtained by means of the mutational analysis of PTEN, describe two sites of binding to ATP, which could only interact if the PTEN protein adopted a different shape to the structure of phosphatase classically described. Moreover, the results presented by Lindsay et al, 2006, which demonstrate the lack of mobilization of nuclear IP3 in the presence of PTEN, would point towards a role different to that of phosphatase for this protein. In conclusion, the best explanatory model for our results and those previously published would be the occurrence of a substantial structural change of the PTEN protein, which would preclude its activity as phosphatase and would establish the existence of a role not previously described for this tumor suppressor.

## Materials and Methods

### Peptides and proteins

The targets used were PTEN peptide, Anp32 peptides, and GST-PTEN (amino terminal domain) of 40,000 Daltons. Synthetic peptides, including the sequence corresponding to amino acids 33–47 for human and mouse PTEN phosphatase (33-IAMGFPAERLEGVYR-amide-47, show in the **Figure 1**), were commercially obtained at an HPLC purity of 70–85% (antigen grade Alpha Diagnostic International, Inc., San Antonio, TX USA). The DNA encoding human PTEN phosphatase amino-terminal domain was obtained by PCR, cloned, then sequenced in the expression vector pGEX2T (Amersham Pharmacia Biotech, NJ, USA). Protein was obtained by transformation of *Escherichia coli*, and its expression was induced using isopropyl thiogalactoside. Pure proteins were obtained using a glutathione-Sepharose column (Glutathione Sepharose-4 fast-flow, Amersham Biosciences, Uppsala, Sweden).

### Aptamer design and selection

#### Library construction

An 87-nucleotide synthetic DNA composed of 21 and 16 nucleotides flanking a “variant region” of 50 random nucleotides (5′-CGGAATTCCGAGCGTGGGCGT (N)_50_ TACGCCCACGCTCGAG-3′ (87 mer)), was generated and purified by HPLC (Integrated DNA Technologies Inc., Coralville, IA, USA). This target sequence was amplified using primer 1: 5′-CGGAATTCCGAGCGTGGGCGT-3′ (21 mer), and primer 2: 5′-CTCGAGCGTGGGCGTA-3′ (16 mer). Biotin was used to label the primers at the 5′- ends (Integrated DNA Technologies Inc., Coralville, IA, USA). The extremes of the constant sequence (underlined in the library sequence) of the aptamers were complementary, capable of producing a double helix of 13 bases. The variable region was where the binding was expected to take place. Each tube contained 100 µl of PCR mixture with 4.2 pmoles (400 nM) of combinatorial library, containing approximately 4.0×10^13^ different molecules. The PCR mixture (Tris 10 mM pH 9 at 25°C, KCl 50 mM, Triton X-100 0.1%, dNTP 1 mM, MgCl_2_ 1.5 mM, each primer at 1 µM) was cycled at 94°C for 30 sec in the denaturing step, at 58°C for 30 sec in the annealing step, and at 72°C for 10 sec in the elongation step. Cycle numbers are indicated in each experiment. Double chain products of PCR were separated by heating at 95°C (5 min) and then cooled with two volumes of PBS/3% of BSA and maintained on ice until use.

#### Target presentation

The phosphatase domain is the most highly conserved region of the PTEN protein and was chosen as the target for aptamer selection. Two regions of the phosphatase were used as sequential epitopes of the target: one a synthetic peptide comprising the 33–47 residues of PTEN and the second the entire amino terminal domain of human PTEN (200 amino acids) in recombination with the carboxy terminal of the glutathione-S-transferase (GST-PTEN). This target was denatured previously to be used for oligonucleotide selection, in order to obtain sequential epitopes from the protein.

Both targets, peptides and proteins, were presented on a physical support of nitrocellulose to allow interaction with the single-strand library of oligonucleotides. The membrane containing the target protein was incubated with 200 µl of PBS containing 2% BSA and 100 µl of single strands of the PCR amplified library (1∶2) overnight at 4°C or otherwise for 3 hours at room temperature.

#### Partition of the bound oligonucleotides

After target presentation, the bound aptamers were separated from the mixture by washing the nitrocellulose support in 2% BSA-PBS (500 µl) three times for 15 minutes, followed by three 15-minute washes in PBS.

#### Elution

The bound DNA was separated from the target proteins captured on the nitrocellulose membrane (0.125 cm^2^) using 100 µl of milliQ-filtered water, and heating at 95°C for 3–5 min. The eluted DNA was then transferred to another tube, and 5 µl was used as a template for PCR. The mixture and the clones of each aptamer obtained were analyzed by dot blot assay to confirm the target recognition, using the recombinant GST-amino terminal of PTEN protein or the synthetic peptide.

### Dot blot assays

Aptamers with little or no cross-reactivities between the two proteins were identified and selected for use in further studies. Dot blot assays with the aptamers were performed as follows: 200 ng of peptide and 2 µg of recombinant protein GST-PTEN were seeded as drops in a nitrocellulose membrane strip (PROTRAN™; Schleicher & Schuell Inc., Keene, NH), dried and blocked with PBS-BSA 2% prior to the incubation with 1.5 ml of 0.5 µM oligonucleotides in a plastic tube. The 5′-biotin of the oligonucleotides was developed with an alkaline phosphatase system.

### Quantitative analysis by real-time PCR

Quantification of ligand-bound DNA molecules was performed with real-time PCR using the ABI Prism 7500 Sequence Detection System (PE Applied Biosystems, Foster City, CA, USA). PCR analyses were performed in 50 µl reactions with SYBR Green dye (PE Applied Biosystems) and ROX dye as an internal fluorescent reference (Molecular Probes, Invitrogen, Carlsbad, CA, USA). None of the primer sets used gave a signal (*C*_T_>25) in control reactions without templates. Standard curves between 10^9^ and 10^5^ copies were determined for the primer set by dilution of input DNA. The inverse and complementary sequences of primers (13 bases) contained in the library structure were expected to interfere in the efficiency of the polymerase amplification. The synthetic PTENz14 aptamer was used as a template, yielding a slope close to the theoretical value for amplification per cycle (Ct = −3.364 *(log C0)+43.909; R^2^ = 0.983 Efficiency = 98.266%). The number of aptamers in the input was calculated from the cycle thresholds (*C*_T_) for each primer set by extrapolation with the standard curves made using 7500 System Software (Applied Biosystems, Foster City, CA, USA).

### Radioactive labeling of aptamers

Ligands were labeled by adding 1 µl of [^32^P]-dCTP (3,000 Ci/mmole, Amersham Biosciences, UK) to 100 µl of mixture used in the PCR (10 µCi or 370 kBq). The PCR products were separated from the free nucleotide [^32^P]-dCTP using size exclusion G-25 columns (MicroSpin S-200 HR columns, Pharmacia Biotech, USA). Approximately 2.5×10^6^ cpm was incorporated at 1 µM concentration for each 50 µl of PCR reaction.

#### Filter binding assay

Nitrocellulose membranes were used to quantify the ligand-receptor complexes formed between the selected oligonucleotides amplified by PCR and the pure protein at a constant concentration of 0.5 mg/ml. The mixture was incubated for 1 h in a final volume of 40 µl with 50 mM Tris buffer, later partitioned using a dot blot vacuum system, so that the unbound species could be removed by three repeated washes with 250 µl of PBS. The bound oligonucleotide chains were then quantified using real-time PCR and detected by radioactivity using a PhosphorImager screen (Amersham Pharmacia Biotech, NJ, USA). The retained radioactivity was cut off and measured in a liquid scintillation counter (Beckman).

### Ligand oligonucleotide cloning and sequencing

PCR amplification (up to 25 cycles) was applied to 5% of the eluted single-stranded DNA from the nitrocellulose membrane. The 87-bp products from PCR were cloned into an *E. coli* pGEM vector (Vector-T Easy system, Promega Corporation, Madison, WI, USA) and sequenced. The pure plasmid was used as a PCR template (20 ng) using modified primers.

### Aptamer sequences

PTENz7: 5′-CGGAATTCCGAGCGTGGGCGTGGTCATACCGCGCCTATCGAACTCGCCA-CTCGCGTGCAGCTCTGTGTAGGTACGCCCACGCTCGAG-3′

PTENz14: 5′-CGGAATTCCGAGCGTGGGCGTGAGCCCTAAACACAAGTCCGCAGGGGT-GTGGTAATATTCGCAGTTGTGTGTACGCCCACGCTCGAG-3′

### Scatchard analysis

Affinity of ligands for a specific target was determined using the radiolabeled oligonucleotides from the filter binding assays, and the radioactivity was measured with a Beckman scintillation counter. The results obtained for aptamers, mixtures, or clones were used to perform a Scatchard analysis (Prism 4 program from http://www.graphpad.com/prism/Prism.htm).

### Experimental animals

Mice of the inbred strain *Kcnj6wv* B6CBACa *Aw-J*/*A*-*Kcnj6wv* were obtained from Jackson Laboratories (Bar Harbor, ME, USA), and were raised at the Institute of Biotechnological Research, National University of San Martín, Argentina. All experiments were performed in triplicate using adult animals. Animals were sacrificed by decapitation. All animal procedures were performed according to the rules and standards of animal protection laws and the regulations for the use of laboratory animals of the National Institutes of Health, USA. This paper has been evaluated and approved by the Ethics Committee of the Hospital de Clínicas of the University of Buenos Aires, as stated in report number 0298/10 dated April 23rd 2010.

### Antibodies

Commercial antibodies were used to detect the carboxy terminal end of PTEN (human or mouse origin) (1∶50 for histology, clone 6H2.1, Cascade, MA, USA), the amino terminal end of PTEN (PTEN N-19 sc-6818, goat polyclonal, Santa Cruz Biotech., CA) and rabbit carboxy terminal (1∶400) (PTEN C-20 sc-6817-R Santa Cruz Biotech., CA). The PTEN antiserum against the peptide, previously developed in our laboratory, was diluted with PBS-BSA 2%, 1∶50 for immunohistochemistry and 1∶400 for western blot as described Perandones et al 2004 [36].

### Western blot

Tissues were dissected and homogenized in 1∶10 w/v of radio-immunoprecipitation-assay buffer (RIPA) (Tris-HCl 50 mM, pH: 7.5, NaCl 150 mM, Nonidet P40: 1%, deoxycholate: 0.5% and SDS 0.1%). Fifty micrograms of protein was loaded in each lane of a 10% SDS-PAGE gel. The nitrocellulose membranes with the transferred proteins were blocked with PBS-BSA 2% and incubated with antibodies or the oligonucleotides containing biotin label. The antibodies were revealed using alkaline phosphatase linked to secondary antibodies, and the oligonucleotides were revealed using avidin-alkaline phosphatase (Promega Corporation, Madison, WI, USA) or peroxidase systems, as indicated. PTEN amino terminal rabbit polyclonal antibodies were diluted 1∶400 with PBS-BSA 5%. A monoclonal anti-rabbit gamma chain-specific antibody coupled to alkaline phosphatase (dilution 1∶10,000, Clone RG-96, purified antibody, Sigma Co., St. Louis, MO, USA) was used in western blots and developed with color substrate 5-bromo-4-chloro-3-indolyl-phosphate/nitro blue tetrazolium. A commercial goat antibody against PTEN amino-terminal protein was used as a control (dilution 1∶500, sc-6818, Santa Cruz Biotech., CA, USA) and developed with anti-goat IgG coupled with alkaline phosphatase (dilution. 1∶1000, anti-goat IgG-AP, sc-2022, Santa Cruz Biotech., CA, USA). The carboxy domain of PTEN antibody was used in western blot of the subcellular fractionation for measurement of PTEN and developed with a secondary antibody and a chemiluminescent substrate for peroxidase (SuperSignal ®, Thermo Fisher Scientific, USA).

### Histochemistry

Wild-type mouse cerebella were dissected at different postnatal ages, fixed in alcohol/acetic acid (95%/5%) or paraformaldehyde (4%) as indicated, embedded in paraffin, sliced, and mounted on silanized glass (Silane, Sigma Co., St. Louis, MO, USA). The paraffin was removed from the slice and tissues were incubated overnight at 4°C with one of the following: rabbit/goat polyclonal antibodies, or mouse monoclonal antibody or aptamers. Then excess molecules were rinsed away twice with PBS, and developed for 1 h using avidin-peroxidase (1∶10,000, Thermo Fisher Scientific Inc., IL, USA) or secondary antibodies coupled to peroxidase or synthetic aptamers labeled with Cy3 and FITC fluorochromes. After 1 hour of incubation, the avidin excess was removed by three washes of PBS, and later the peroxidase was developed with di-amino-benzidine 8% w/v, H_2_O_2_ 0.3% v/v in PBS buffer. Images were obtained using a BX-60 Olympus IX-71 microscope (Olympus, Japan), digitized with an Optronics camera, and images were merged using the programs Image-J-Pro 4.0 or the Adobe PhotoShop program.

### Comparisons of histologic samples with different aptamers

After blocking the slices, they were incubated overnight at a temperature of 4°C with aptamers containing biotin obtained through PCR. After the amplification, the mixture was heated for 3 minutes to 94°C and then quickly cooled to 4°C by diluting the solution with 2 volumes of cold PBS-BSA 5%. The cold temperature stabilizes the double helix in the solution, formed by the constant sequence of aptamers. Where the synthetic aptamers containing biotin, PTENz7 and PTENz14 (1 µM), were used, the slices were incubated in PBS-BSA 5% for 3 hours at room temperature. As a negative control, slices were incubated with a polyclonal antibody (1∶1,000 in PBS-BSA 5%). A positive control was established using a synthetic aptamer that recognizes protein Anp32e (1 µM).

### Competition with recombinant protein

Histologic samples similar to the ones described above were co-incubated with the aptamers containing biotin (250 nM) and the recombinant protein GST-PTEN (525 µg/ml) for 3 hours at room temperature. As a positive control, slices were incubated with 250 nM of PTENz7 or PTENz14 in PBS-BSA 5% without competition. Finally, the control of the competition with GST-PTEN was carried out using the synthetic aptamer containing biotin that recognizes the protein Anp32a.

#### Blocking with antibodies

In order to better demonstrate the specificity of the aptamers towards phosphatase PTEN, competitions with antiserum from rabbit immunized with recombinant PTEN were performed. After removing the paraffin and blocking the slices with PBS-BSA 5%, they were incubated overnight with the rabbit antiserum and BSA separately in a humidity chamber at 4°C. The pre-incubated slices were incubated with the synthetic aptamers containing biotin for 3 hours at room temperature, again in a humidity chamber. After incubation, 3×10 minute washes were performed with PBS. The aptamers were revealed as described above.

### Competition assays between aptamers and antibodies for recognition of the PTEN peptide

To determine the antigenicity of the epitopes recognized by each selected aptamer, a union assay between aptamers, antiserum and the peptide was carried out in solution in quadruplicate.

#### A) Antibody preparation

An 800 µg sample of protein A coupled to Sepharose (Sigma-Aldrich, USA) was incubated with 2.8 mg of complete proteins obtained from peptide-immunized rabbit serum, in PBS solvent in a final volume of 130 µl. Once the incubation time had elapsed, the solution was centrifuged for 1 minute, ultimately discarding excess supernatant. In order to wash out the immunoglobulin that failed to attach to protein A, the Sepharose beads were washed three times in 130 µl PBS for 5 minutes at room temperature and centrifuged. The pellets were suspended in 130 µl PBS-BSA 1% and afterwards divided into aliquots.

#### B) Attachment of antibodies to peptides

The aliquots were incubated with the peptide (3 µM) at room temperature and agitated for 1 hour to allow the union of the immunoglobulins. Next, a 10-second centrifugation was performed, excess supernatant was discarded and three washes with PBS were completed. Both mixtures, the incubated and the non-incubated peptides, were divided into 2 aliquots each.

#### C) Assays of aptamer union

Each sample containing the Sepharose bead aliquots was incubated with each synthetic aptamer, PTENz7 and PTENz14 (1 µM), separately. The incubation took 1 hour at room temperature with gentle agitation. Each sample was washed three times with 500 µl of PBS following incubation and the bound aptamers were eluted using 20 µl of NaOH 0.15 M, neutralized with 20 µl of HCl 0.15 N.

#### D) Quantification of the eluted aptamers

The quantification of the aptamers obtained was performed by real-time PCR, as described above in the section on Aptamer Selection. From each aptamer sample 2 µl aliquots were quantified, in quadruplicate, using 35 cycles at 94°C for 30 sec in the denaturing step, at 58°C for 30 sec in the annealing step, and at 72°C for 10 sec in the elongation step.

### Antigen retrieval

Paraffin was removed from the slices, which were later hydrated in citric acid (10 mM pH 6.0) and incubated for 20 minutes at 95°C in order to denature the proteins. Three washes with PBS were performed for 2 minutes and slices were later blocked with PBS-BSA 5%.

### Precipitation with aptamers

#### Precipitation of PTEN

The cerebellum homogenate was divided into 500 µl aliquots. To prevent the unspecific interaction between protein and aptamers, 12 µg of fragmented salmon sperm was added to each aliquot. The mixture was transferred to a tube that contained the equivalent to 150 µl of Streptavidin-magnetic microspheres (Streptavidin MagneSphere Paramagnetic Particles, Promega, WI, USA) in order to absorb the nonspecific proteins. After incubating the mixture for 30 minutes at room temperature with gentle agitation, the magnetic particles were removed. The supernatants were incubated with the synthetic aptamers: PTENz7, 250 nM; PTENz14, 250 nM and Anp32a (250 nM), or lysozyme reverse aptamers as negative controls. The Streptavidin-magnetic microspheres were added once more to the mixture and incubated for 1 hour at room temperature with gentle agitation. The microspheres were separated from the homogenate mixture and washed with 500 µl of RIPA (1∶2) for 5 minutes, then washed twice with PBS. The proteins retained by the aptamers were recovered with 10 µl of NaOH 0.15 M and immediately neutralized with 10 µl of HCl 0.15 M. The proteins obtained were used in western blot assays by means of goat antibodies against the amino terminal of PTEN (sc-6818, Santa Cruz Biotech., CA, USA).
